# Between practice and evidence: the Revista Brasileira de Medicina do Trabalho paths in search of excellence

**DOI:** 10.47626/1679-4435-2023-211

**Published:** 2023-04-18

**Authors:** Mírian Perpétua Palha Dias Parente

**Affiliations:** Editor-in-chief, Brazilian Journal of Occupational Medicine

In a recent editorial,^1^ we have shared the paths traveled and advances made by the Brazilian Journal of Occupational Medicine (Revista Brasileira de Medicina do Trabalho, RBMT) in the last three years.

Now, with the beginning of a new management of the Brazilian National Association of Occupational Medicine (Associação Nacional de Medicina do Trabalho, ANAMT) and, consequently, of RBMT, and open to readers’ suggestions, we present our intentions for the coming years.

Actions have been proposed to improve the RBMT in order to increase the journal’s citation rates (impact factor, h-index, and CiteScore, among others), and will be progressively implemented, such as:

- Consent for the submission of articles previously deposited in non-commercial preprint repositories;
- Adoption of the continuous publication system;
- Restructuring of the Editorial Board, including more members with relevant publications who may contribute suggestions to improve the journal;
- Continuous improvement of instructions for authors, allowing the submission of manuscripts with more relevant content while meeting the challenging database requirements;
- Increased participation of international reviewers and authors;
- Training of reviewers through online courses in the format of “Continuing Medical Education,” always promoting good practices and seeking to strengthen ethics in scientific publication;
- Provision of templates in the publication management system Sistema de Gestão de Publicações (SGP) for responding to peer reviewer comments, thus improving and standardizing the review process;
- Holding frequent regular meetings of the Editorial Board for evaluation and planning;
- Content direction on social media, sending texts of Occupational Medicine interest to be shared and choosing the articles to be prioritized on social media;
- Request for indexing in the Directory of Open Access Journals (DOAJ) and PubMed; and
- Inclusion of a form to be completed by the authors informing about compliance of the manuscript with Open Science communication practices, as adherence to them “makes research more efficient, reliable, and creative.”^2^

Since December 2022, accepted articles have been made available on our website as *Epub ahead of print*, with no publication date defined but with a DOI assigned to them, allowing them to be shared and cited.

Meanwhile, we continue to value and encourage the discussion of topics such as the impact of preprint and the challenges for publications with the increasing use of artificial intelligence and its ethical conflicts, among others.

The challenges are massive, but nothing will keep us from doing our best to provide our audience with readings that can enhance their technical-scientific knowledge, always seeking to improve the experience of all those dedicated to this fascinating field of Occupational Medicine.

Have a good read!
